# SARS-CoV-2 Infections and Viral Isolations among Serially Tested Cats and Dogs in Households with Infected Owners in Texas, USA

**DOI:** 10.3390/v13050938

**Published:** 2021-05-19

**Authors:** Sarah A. Hamer, Alex Pauvolid-Corrêa, Italo B. Zecca, Edward Davila, Lisa D. Auckland, Christopher M. Roundy, Wendy Tang, Mia Kim Torchetti, Mary Lea Killian, Melinda Jenkins-Moore, Katie Mozingo, Yao Akpalu, Ria R. Ghai, Jessica R. Spengler, Casey Barton Behravesh, Rebecca S. B. Fischer, Gabriel L. Hamer

**Affiliations:** 1 College of Veterinary Medicine and Biomedical Sciences, Texas A&M University, College Station, TX 77843, USA; pauvolid@cvm.tamu.edu (A.P.-C.); ibzecca@cvm.tamu.edu (I.B.Z.); edavila@tamu.edu (E.D.); lauckland@cvm.tamu.edu (L.D.A.); 2 Laboratory of Respiratory Viruses and Measles, SARS-CoV-2 National Reference Laboratory and Regional Reference Laboratory in the Americas (PAHO/WHO), Fiocruz, Rio de Janeiro 21040-360, Brazil; 3 Department of Entomology, Texas A&M University and AgriLife Research, College Station, TX 77843, USA; cmroundy@tamu.edu (C.M.R.); wtang@tamu.edu (W.T.); ghamer@tamu.edu (G.L.H.); 4 National Veterinary Services Laboratories, USDA APHIS VS, Ames, IA 50010, USA; mia.kim.torchetti@usda.gov (M.K.T.); mary.l.killian@usda.gov (M.L.K.); melinda.jenkins-moore@usda.gov (M.J.-M.); katherine.m.mozingo@usda.gov (K.M.); 5 Brazos County Health Department, Bryan, TX 77803, USA; YAkpalu@brazoscountytx.gov; 6 National Center for Emerging and Zoonotic Infectious Diseases, U.S. Centers for Disease Control and Prevention, Atlanta, GA 30329, USA; ofu9@cdc.gov (R.R.G.); wsk7@cdc.gov (J.R.S.); dlx9@cdc.gov (C.B.B.); 7 School of Public Health, Texas A&M University, College Station, TX 77843, USA; rfischer@tamu.edu

**Keywords:** SARS-CoV-2, cats, dogs, reverse zoonosis, virus isolation, neutralizing antibodies

## Abstract

Understanding the ecological and epidemiological roles of pets in the transmission of SARS-CoV-2 is critical for animal and human health, identifying household reservoirs, and predicting the potential enzootic maintenance of the virus. We conducted a longitudinal household transmission study of 76 dogs and cats living with at least one SARS-CoV-2-infected human in Texas and found that 17 pets from 25.6% of 39 households met the national case definition for SARS-CoV-2 infections in animals. This includes three out of seventeen (17.6%) cats and one out of fifty-nine (1.7%) dogs that were positive by RT-PCR and sequencing, with the virus successfully isolated from the respiratory swabs of one cat and one dog. Whole-genome sequences of SARS-CoV-2 obtained from all four PCR-positive animals were unique variants grouping with genomes circulating among people with COVID-19 in Texas. Re-sampling showed persistence of viral RNA for at least 25 d-post initial test. Additionally, seven out of sixteen (43.8%) cats and seven out of fifty-nine (11.9%) dogs harbored SARS-CoV-2 neutralizing antibodies upon initial sampling, with relatively stable or increasing titers over the 2–3 months of follow-up and no evidence of seroreversion. The majority (82.4%) of infected pets were asymptomatic. ‘Reverse zoonotic’ transmission of SARS-CoV-2 from infected people to animals may occur more frequently than recognized.

## 1. Introduction

The pandemic of coronavirus disease 2019 (COVID-19), caused by the novel severe acute respiratory syndrome virus 2 (SARS-CoV-2), is an unprecedented challenge to health, the economy, and nearly all aspects of society. Phylogenetic studies that reconstruct the evolutionary relationships between SARS-CoV-2 and its closest relatives suggest the reservoir of SARS-CoV-2 was an animal host, likely horseshoe bats (*Rhinolophus* spp.). However, the virus may have emerged in the human population by way of a yet unidentified intermediate host [1]. As the pandemic continues, studies have established that several mammalian groups, including primates, felids, mustelids, and some species of rodent, lagomorph, and bat, are susceptible to natural or experimental infection [2]. Across the globe, there are two common groups where suspected human-to-animal transmission events have been repeatedly detected: farmed mink and companion animals. Outbreaks have occurred on mink farms in the Netherlands, Denmark, Spain, Italy, Sweden, Greece, and the United States between April and November 2020. Outbreaks in Europe led to widespread culling and moratoria being placed on the mink industry [3]. With respect to companion animals, over 10 countries to date have documented natural infections of dogs and cats, often associated with exposure to a person with COVID-19 [4]. For example, the first cases of companion animal infection with SARS-CoV-2 in the United States were two cats in New York that developed respiratory signs after exposure to their owners with suspected or confirmed COVID-19 [5].

Experimentally, cats have been shown to be highly susceptible to infection by SARS-CoV-2 and can transmit the virus to other cats under laboratory conditions via both direct and indirect contact [6,7,8,9]. Bosco-Lauth et al. [8] demonstrated that neutralizing antibodies protected cats against subsequent challenges. There is also evidence from laboratory challenge studies that dogs have lower susceptibility with limited viral replication, although seroconversion with neutralizing antibodies has been confirmed for both dogs and cats [7,8].

Despite the potential public health and animal health implications of SARS-CoV-2 infections in companion animals, few epidemiological investigations of companion animals living in household environments are available. Most of the evidence for natural animal exposures and infections comes from opportunistic case studies [10,11], the testing of animals presenting for routine veterinary visits, or studies in congregate animal settings [12]. Focused studies on animals with known exposure to people with COVID-19 have the potential to quantify the probability of infection in animals that have sustained contact with an infected person. They, therefore, are critical in understanding the potential for companion animals to serve as reservoirs for the virus. The objective of this study was to establish an epizootiological investigation program based on the active surveillance of dogs and cats from households with SARS-CoV-2-infected owners in order to quantify the prevalence of domestic animal infection in these high-risk natural environments in Texas, a state with high numbers of reported human cases in summer 2020 [13]. 

## 2. Materials and Methods

### 2.1. Animal Recruiting and Sampling

The study enrollment criteria included any dog or cat living in the same household as a person with a confirmed (PCR-positive) SARS-CoV-2 infection; no restrictions were made based on breed, age, vaccination status, or medical history of the animals. Individuals that tested positive for SARS-CoV-2 were contacted via phone by the Brazos County Health Department (BCHD) as part of a public health case investigation. Individuals were asked if they owned pet dogs or cats, and if they did, if they wished to learn more about enrolling their pets in a Texas A&M University (TAMU) research project sampling animals for SARS-CoV-2. Interested pet owners were provided the project website (tx.ag/BCSCovidResearch), and the contact information of the consenting pet owners was provided to the TAMU investigation team. Pet owners were administered a short questionnaire by phone including pet signalment (breed, age, sex), vaccination history, pet symptoms, date of positive human test result (which was cross-checked with BCHD), and were read the details of the informed consent form, after which a visit was arranged to sample the animal(s) at their household.

All samples were obtained from privately-owned animals in accordance with the relevant guidelines and regulations approved by the TAMU’s Institutional Animal Care and Use Committee and Clinical Research Review Committee on May 14, 2020 (2018-0460 CA). Written consent was received for each pet from the owner. The Institutional Review Board issued a determination that this study is exempt from human subject research review. For sample collection at the household, the TAMU research team was deployed to the homes and collected three swab samples from each animal: (i) respiratory; (ii) rectal; and (iii) external body (fur). The respiratory sample was a combination of an oral/oropharyngeal swab, a nasal/nasopharyngeal swab, and a conjunctival swab (cats only). The rectal sample included a single swab inserted up to 1.5 cm in the rectum and around the external surface. Due to concerns that emerged early in the pandemic of animals serving as fomites for the virus, body (fur) swabs were also collected by rubbing two swabs over the animal’s scruff, ears, neck, back, and abdomen. All of the swabs used were 5.2 mm diameter standard polyester tipped applicators with polystyrene handles (Puritan Medical Products, Guilford, ME, USA) except for cat and small dog respiratory samples, where 3.3 mm diameter polyester swabs with propylene handles (Constix^®^, Contec, Spartanburg, SC, USA) were used. Swabs were submerged immediately into 3 mL of viral transport media (VTM; made following CDC SOP#: DSR-052-02) and left in the media until aliquots were prepared within 24 h. Blood was collected via cephalic, jugular, or medial saphenous venipuncture into clot activator and EDTA tubes. All samples were kept in a cooler with ice packs until returned to the lab. Swabs were placed in a −80 °C freezer. Blood samples were centrifuged, and aliquots of serum, clot, whole blood, plasma, and red blood cells were prepared, which were stored at −80 °C. 

For households with animals that tested positive (via detection of SARS-CoV-2 viral RNA or antibodies), efforts were made to longitudinally re-sample all animals living in that household regardless of the initial testing result of each individual animal. Repeat specimen collections followed a minimum of one week from the date of initial sample collection and occurred up to three times per household within three months of the first specimen collection event. At each follow-up visit, identical sets of specimen types were collected, and owners were asked about any changes in their pet(s) health. For some follow-up events, an updated protocol was implemented, which included the collection of oral/oropharyngeal, nasal/nasopharyngeal, and conjunctival swabs separately, rather than combining these swabs into a single respiratory sample. 

### 2.2. Molecular Testing

All specimens were evaluated for the presence of SARS-CoV-2 viral RNA at TAMU using the following protocols. The VTM was homogenized, and a 400 μL aliquot was removed. Viral RNA was extracted using a MagMAX CORE Nucleic Acid Purification Kit on a 96-well Kingfisher Flex System (ThermoFisher Scientific, Waltham, MA, USA). A subset of samples was tested for RNA concentration on an Epoch Microplate Spectrophotometer (BioTek, Winooski, VT, USA). All samples were screened on two separate qRT-PCRs targeting the RNA-dependent RNA polymerase (RdRp) gene and the E gene [14,15]. Briefly, a 25 μL reaction included 5 μL of sample RNA, 6.25 μL of 4× RT-Buffer (TaqMan Fast Virus 1-Step Master Mix, ThermoFisher Scientific), 600 nM of the forward primer (RdRp_SARSr-F), 800 nM of the reverse primer (RdRp_SARSr-R), and 100 nM of the probe (RdRp_SARSr-P2); a control plasmid containing a portion of the RdRp gene served as a positive control. The E gene 25 μL reaction consisted of 5 μL of viral RNA, 6.25 μL of 4× RT-Buffer, 400 nM of the forward primer (E_Sarbeco_F), 400 nM of the reverse primer (E_Sarbeco_R), and 200 nM of the probe (E_Sarbeco_P1); a control plasmid containing the complete envelope gene served as a positive control. All primers, probes, and positive controls were from IDT (Integrated DNA Technologies, Coralville, IA, USA). Both reactions followed the previously published thermocycling conditions [14,15], which consisted of 50 °C for 30 min for reverse transcription, followed by 95 °C for 15 min for RT inactivation/initial denaturation, then 45 cycles of 95 °C for 15 s, and 58 °C for 60 s using a CFX96 Real-Time System (BIORAD, Hercules, CA, USA). Negative controls were included in the nucleic acid purification plates (VTM) and qPCR plates (PCR-grade water) with no indication of contamination in the study.

All specimens that were non-negative using either protocol were submitted for confirmatory PCR testing at the USDA National Veterinary Services Laboratory (NVSL) with joint approval from the Texas Department of State Health Services and the Texas Animal Health Commission. Confirmation of sample status in the national laboratory is a requirement for the case definition of animal SARS-CoV-2 infection. An 800 μL aliquot of the original VTM swab sample was sent to NVSL where RNA was extracted, and two additional qRT-PCR protocols were followed, targeting virus nucleocapsid gene region 1 (N1) and nucleocapsid gene region 2 (N2) for specific detection of SARS-CoV-2 following the CDC’s protocol [16]. Additionally, partial and whole-genome sequencing was attempted for any positive sample using RNA extracted directly from the diagnostic samples [17]. For some swabs from longitudinally-sampled animals, the initial screening PCRs were conducted at the Wisconsin Veterinary Diagnostic Laboratory, followed by confirmatory testing at NVSL for any non-negative samples.

To explore other coronaviruses that may be present in the sampled animals, all respiratory swabs collected initially from each animal were subjected to a conventional RT-PCR to amplify a 668 bp-region within the RdRp gene that encodes the most conserved protein domain of α-, β-, γ-, and δ-coronaviruses [18]. Amplicons were purified and submitted for bi-directional sequencing (Eton Biosciences, San Diego, CA, USA), followed by manual editing and submission to Genbank. This step was done to screen for additional coronaviruses present in the study population, which could inform the viral and serological diagnostics.

### 2.3. Viral Isolation and Plaque Assay

Specimens considered positive by qRT-PCR were subjected to virus isolation as described previously [19]. Briefly, at the NVSL, the samples were diluted from 1:2 to 1:3 in minimum essential medium with Earle’s balanced salt solution (MEM-E). Vero (ATCC CCL-81) cells were inoculated with 1.5 mL of diluted sample and adsorbed for 1 h at 37 °C. After adsorption, a replacement medium was added, and cells were incubated at 37 °C for up to seven days. Cell cultures with no CPE were frozen, thawed, and subjected to two blind passages, with inoculation of the fresh cultures with the lysates as described above. Viral infection was confirmed through reduction of the Ct values in the cell cultures with SARS-CoV-2-specific qRT-PCR using the CDC N1 and N2 primer and probe sets. Independently, samples positive by qRT-PCR were also subjected to virus isolation efforts at TAMU. First, samples were diluted at 1:2 in Medium 199 (Gibco) supplemented with 5% FBS and then inoculated in Vero CCL-81 cells as described. Following inoculation, adsorption, and media replacement, cells were incubated with Medium 199, 5% FBS and 1% penicillin-streptomycin-antimycotic at 37 °C for 4 days. All samples were passaged a total of three times by being frozen and thawed before inoculation of the fresh cultures with the lysates as described above. Aliquots from each passage were collected for RNA extraction and qPCR. For plaque assays, at the TAMU laboratory, the qRT-PCR-positive samples that were positive for viral isolation were serially diluted from 10^−1^ to 10^−6^ in Medium 199 supplemented with 5% FBS. A total volume of 200 uL was inoculated in duplicates to Vero 6-well plates and incubated at 37 °C and 5% CO_2_ for 1 h. After incubation, a 0.5% agarose overlay medium was added to each well with infected cells. Following a 3-day incubation period, a secondary overlay solution containing neutral red was added to each well, and plates were incubated for an additional day. On the fourth day post-infection, plaques were counted on a lightbox.

### 2.4. Whole Genome Sequencing and Phylogenetic Analysis

Libraries for whole-genome sequencing were generated using the Ion AmpliSeq Kit for Chef DL8 and Ion AmpliSeq SARS-CoV-2 Research Panel (Thermo Scientific, Waltham, MA, USA). Libraries were sequenced using an Ion 520 chip on the Ion S5 system using the Ion 510™ & Ion 520™ & Ion 530™ Kit. Sequences were assembled using IRMA v. 0.6.7 [20] and visually verified using DNAStar SeqMan NGen v. 14. MAFFT [21,22] was used to align FASTA files using a maximum iterative refinement number of 1000. The alignment was used to output a phylogenetic tree using RAxML with the GTRCAT model [23]. The tree was rooted with the Wuhan seafood market reference genome (NC_0445512) and additional whole-genome SARS-CoV-2 sequences from felines and canines across the world. Sequences from infected humans in Texas were retrieved from NCBI and added to the analysis. Full genomes generated in this study were submitted to Genbank (accession nos. MW263334-7).

### 2.5. Virus Neutralization Assay

All serum samples were assayed for SARS-CoV-2 neutralizing antibodies at NVSL through virus neutralization (VN). VN was used for screening as ELISA assays for animal studies were not established when this study began. For VN, 25 µL of two-fold serially diluted sera (for final dilutions with the virus of 1:8 to 1:2048) were pre-incubated with 25 µL of 100 TCID_50_/_mL_ of SARS-CoV-2 (2019-nCoV/USA-WA1/2020; ATCC NR-52281) in MEM-E containing 200 UI/mL penicillin, 200 µg/mL streptomycin, 75 µg/mL gentamicin sulfate and 6 µg/mL Amphotericin B for 60 min at 37 °C with 5% CO_2_. Each serum sample was tested in duplicate in 96-well plates. At 1 h post-infection, 150 μL of Vero 76 cell (ATCC CRL-1587) suspension was added to the virus-serum mixtures. The neutralization titers were determined at 3 days post-infection. The titer of a sample was recorded as the reciprocal of the highest serum dilution that provided at least 100% neutralization of the reference virus, as determined by visualization of CPE. The specificity of the VN assay was assessed in-house by testing sera from naturally infected animals with antibodies to transmissible gastroenteritis virus (TGEV), porcine epidemic diarrhea virus, porcine hemagglutinating encephalomyelitis virus, bovine coronavirus (BCoV), and Aleutian disease. Additional specificity testing was conducted at a partner laboratory by testing sera with antibodies to common feline and canine coronaviruses. The TGEV was selected as a canine/feline coronavirus surrogate since all these viruses belong to the Alphacoronavirus-1 species and BCoV was used as a canine respiratory coronavirus surrogate since they both belong to the Betacoronavirus-1 species. All of this testing was negative by the VN.

### 2.6. National and International Reporting

The USDA case definition for a confirmed positive case of SARS-CoV-2 in animals includes PCR detection of both N1 and N2 at NVSL and sequence confirmation of the virus either directly from the specimen or from the virus isolate, or demonstration of a SARS-CoV-2 neutralizing antibody at NVSL [24]. Confirmed positive cases were reported to the CDC and the United States Department of Agriculture (USDA) [25]. USDA subsequently reported it to the World Organisation for Animal Health (OIE) [4].

## 3. Results

### 3.1. Demographic Data

Between 24 June–31 July 2020, 76 pets from 39 households were sampled in Brazos County, Texas. Among the 59 dogs, a total of 21 breeds were represented, comprised in the American Kennel Club breed groups as follows: working (24%), terrier (22%), toy (20%), herding (14%), non-sporting (10%), hound (7%), sporting (2%) and Foundation Stock Service (2%). Among the 17 cats, the majority (82.4%) were domestic shorthaired cats, with a minority domestic longhaired (11.8%) and a single Maine coon (5.9%). Cats ranged in age from 3 months to 10 years, and dogs ranged in age from 1.5 months to 18 years. Specimens were collected from pets 3–27 days after the human household member received a positive test result (mean 9 days; median 8 days), with up to three follow-up specimen collections for some pets occurring up to 7 September 2020.

### 3.2. Infection Prevalence and Viral Isolation

SARS-CoV-2 infection was confirmed by PCR and sequencing in 3 out of 17 (17.6%) cats, and 1 out of 59 (1.7%) dogs sampled from 4 out of 39 (10.3%) separate households; these four animals met the USDA animal case definition for SARS-CoV-2 infection, with positive respiratory and/or rectal swabs (Table 1). An additional two dogs that lived together tested positive by rectal and body swabs (TAMU-044) or body swab only (TAMU-043) but did not meet the case definition due to failed sequencing likely due to the presence of a low concentration of viral targets based upon high Ct values. Further, these two dogs had no evidence of neutralizing antibodies and did not develop any upon re-sampling, supporting the absence of prior infection, and that detection by PCR likely reflects living in a contaminated environment.

In one of four SARS-CoV-2 confirmed animals based on PCR/sequence data (cat TAMU-013), all three swab types (respiratory, rectal, and body) tested positive by all PCR tests. Additionally, this same cat was the only animal to test positive in the pan-coronavirus conventional PCR, resulting in an RdRp gene partial sequence with 100% sequence homology to several SARS-CoV-2 isolates from human cases in the United States (Genbank accession no. MT911466). Of the three other confirmed cases based on PCR/sequence data, all had positive respiratory swabs, with one cat also testing positive by PCR via rectal swab and a dog testing positive by PCR from body swabs (Table 1). All four confirmed cases were sampled within 7 days of the reported diagnosis of their owner (Table 1). Of the four households with a confirmed case, the median number of days between a positive human test result and the sampling of pets was 6 days. In contrast, of the 35 households with no pet meeting the case definition based on PCR, the interval was 8 days (Mann-Whitney U test, P = 0.08). 

Virus isolation was attempted from 13 specimens (Table 1), including 10 different specimens from initial or re-sampling of the four animal cases confirmed by sequence, plus three additional specimens from the two animals that were PCR-positive on body swabs. SARS-CoV-2 was initially isolated from the respiratory swab of TAMU-013 on the second passage. Virus recovery was confirmed by PCR (Ct value reduction from original inoculum on N1 and N2 assays from 18 and 17.7 to a cell suspension of the second passage of 15.31 and 15.37, respectively). In independent isolation efforts at TAMU, the virus was isolated from respiratory swabs of TAMU-013 (cat) and TAMU-077 (dog; Table 2). For TAMU-013, CPE was seen on the third day post-infection following a single passage and was confirmed by a reduction in PCR Ct values for the RdRp viral gene and an increase in viral titer by plaque assay (Table 2). For TAMU-077, no CPE or measurable titer by plaque assay was observed from the original sample or following any passage. However, the Ct value for the RdRp gene decreased during passaging (Table 2).

### 3.3. Sequence Analysis

**Figure 1:**
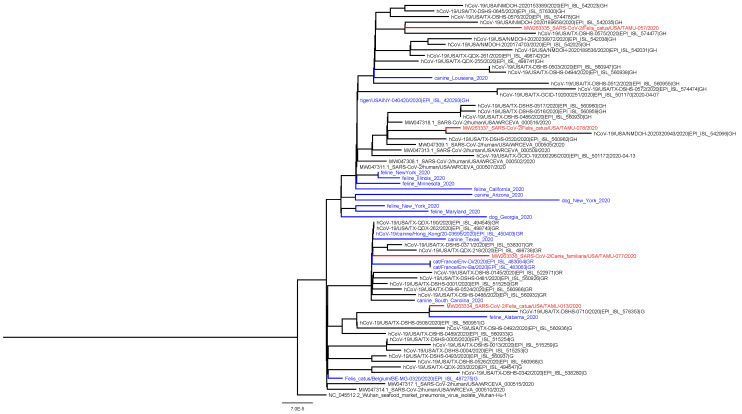
Phylogenetic analysis of SARS-CoV-2 whole genome sequences obtained from this study. These include three cats and one dog (red; animal ID: TAMU-013, 057, 077, 078) and SARS-CoV-2 genomes from feline and canine hosts based on prior studies (blue) and from humans from Texas (black). The analysis used RAxML with the GTRCAT model [23].

A coding-complete genome sequence was obtained for all four confirmed positive animals from respiratory or rectal swabs collected at the first visit (Table 1). All genomes shared high levels of identity to SARS-CoV-2 (99.97–99.98%) and contained 12–16 more mutations compared to reference sequence Wuhan-Hu-1. The animal viruses belong to clades G (*n* = 1), GH (*n* = 2), and GR (*n* = 1),which correspond to the predominant clades observed for human samples in Texas during the same time period (Figure 1). No mutations were observed in the spike protein receptor-binding domains. 

### 3.4. Virus Neutralization and Household Patterns

Viral neutralization was attempted for 75 out of the 76 animals in the study; one cat was fractious at the time of sampling, so no blood was obtained. At baseline specimen collection, 14 out of 75 (18.7%) pets had SARS-CoV-2 neutralizing antibodies, including 7 out of 16 (43.8%) cats and 7 out of 59 (11.9%) dogs from 10 out of 39 (25.6%) households. These included two out of the four (50%) PCR-confirmed positive case animals (Table 3). Virus neutralization titers ranged from 1:16 to 1:128 upon initial sampling.

**Figure 2:**
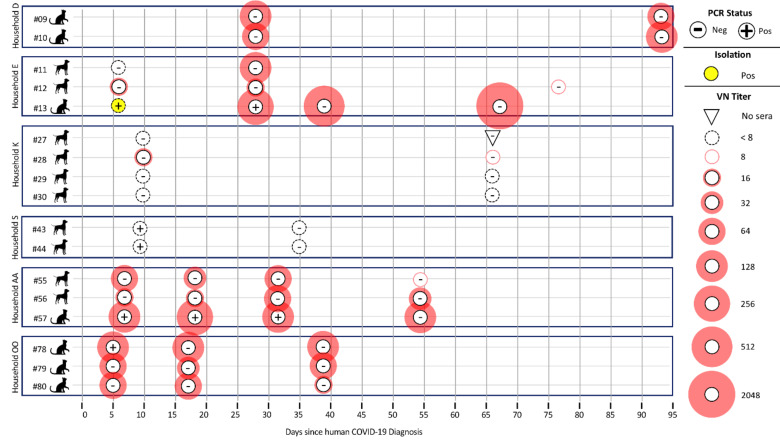
Multi-pet households in which at least one pet in the house was initially positive by PCR analyses or virus neutralization. All pets in the household were followed longitudinally to track duration of positive test results and changes in antibody titers over time. PCR status is based on preliminary screening Ct of <40 on RdRp, E, N1 and/or N2 gene target from any diagnostic swab type (respiratory, rectal, or body swab; positive results here do not necessarily indicate USDA case definition has been met. Virus neutralization titer was determined by a two-fold serial dilution of sera.

Among animals in multi-pet households (Figure 2), there are some instances where all animals were negative by PCR yet positive by VN. For example, both cats from household D were PCR-negative yet VN-positive and were sampled 27 days after owner diagnosis. In household K, all four dogs were PCR-negative, and one was VN-positive, and they were sampled 10 days after owner diagnosis. In the three multi-pet households, each had a single cat test PCR-positive at the initial visit with neutralizing antibodies found in one out of three (household E; sampled 6 days after owner diagnosis) and in three out of three pets at the second sampling (household AA; sampled 7 days after owner diagnosis, and household OO; sampled 5 days after owner diagnosis).

### 3.5. Longitudinal Sampling of Infected Animals

Animals that tested positive by PCR and/or VN, and all other animals living in the household with positive animals, were re-sampled up to three times to describe the natural history of infection in terms of viral RNA and antibody titers over time, resulting in 30 follow-up events. In two out of the four animals initially confirmed by PCR/sequence, the follow-up samplings were negative by PCR (Table 1). However, for one cat (TAMU-013), the body swab remained PCR-positive (for two out of four viral targets) from the second sample acquired 3 weeks later (27 days after the owner’s diagnosis) with comparable Ct values. In one other cat (TAMU-057), the respiratory swab remained positive by PCR (for two out of four viral targets) in the second sample that was acquired 1.5 weeks later but with increased Ct values, and also in the third sampling (for one out of four viral targets) nearly four weeks after the first sample but with a high Ct value. Genome sequence efforts were not successful for these later time points. This represents the longest duration of detection of viral RNA by preliminary screening in a companion animal sample to date, with the last PCR positive result recorded 32 days following the owner’s diagnosis (Table 1).

Among all 15 animals that had sequential serum samples collected, the VN titer values were relatively stable or increased in the 2–3 months of follow-up, with no evidence of seroreversion (Table 2). For example, one cat (TAMU-057), which was also PCR-positive at the initial visit, showed relatively stable VN titers of 1:128, 1:256, 1:128, and 1:128 at four sequential visits from 7–54 days post owner diagnosis. There was also evidence of seroconversion followed by a marked increase in VN titer in some animals. For example, one cat (TAMU-013) was PCR-positive and viral isolation-positive, yet VN-negative, at the first visit, had a VN titer of 1:256 by the second visit 21 days later, and a final titer of 1:2048 by the fourth visit 61 days after the initial visit. 

In examining the multi-pet houses that were re-sampled, we see that the number of VN-positive animals in the house increased over time. For example, household E, where one cat and two dogs live, had an initial sampling status of PCR-positivity in the cat, with only one dog testing VN-positive. By three weeks later, the cat was still PCR-positive, and all three animals were VN-positive. Additional sampling of the cat (TAMU-013) showed seroconversion followed by an increasing VN titer (negative, 1:256, 1:512, and 1:2048 at the four visits, respectively).

### 3.6. Pet Signs of Disease

Among the four pets confirmed by PCR/sequence and two others that were presumptive positive by PCR but were not confirmed, owners reported all pets to be asymptomatic prior to the time of first sampling. Subsequently, one cat (TAMU-013) was reported to sneeze for approximately three days following the first sampling. Additionally, another cat (TAMU-078) was reported to be more sleepy than normal for 2 weeks following the first sampling. Among the additional 13 animals that were VN-positive during initial or re-sample events, a single dog (TAMU-028) was reported to sneeze prior to initial sampling. When re-sampled, all animals were reported to be in good health.

## 4. Discussion

There are few active surveillance studies assessing SARS-CoV-2 infection in companion animals in households with documented human infections. In France, one study of 9 cats and 12 dogs owned by people with COVID-19 showed no evidence of SARS-CoV-2 viral RNA or antibodies [26], while another study of 22 cats and 11 dogs found a single cat (4.5%) positive for SARS-CoV-2 by RT-PCR [27]. A study in Hong Kong, China, documented that 2 out of 15 (13.3%) dogs living in homes with human COVID-19 had SARS-CoV-2 viral RNA in nasal, oral, and rectal swabs and also demonstrated measurable antibody titers on subsequent samples [28]. In another Hong Kong study, 6 out of 50 (12%) cats living with humans with SARS-CoV-2 infection were positive by RT-PCR [29]. 

The current study rapidly deployed a field team to assess infection among pets living with laboratory-confirmed SARS-CoV-2-infected owners and used a comprehensive sampling and testing protocol. We focused sampling on dogs and cats at high risk of exposure to SARS-CoV-2 from their owners at a time when the rate of human community transmission in Texas was high (average of 27.3 cases/100,000 people in the state during the late June-July study period) [13]. Out of 17 cats, one (5.9%) had SARS-CoV-2 isolated in cell culture from a respiratory swab, 16.7% tested positive by PCR/sequencing from one or more swab types, and 41.2% had neutralizing antibodies. Out of 59 dogs, SARS-CoV-2 RNA was detected in sequential cell passages from a respiratory swab from one (1.7%), which was also the only dog to test positive by PCR/sequencing, and 11.9% had neutralizing antibodies. The phylogenetic analysis from this study shows that four of the SARS-CoV-2 genomes recovered from three cats and one dog are of unique lineages and likely represent reverse zoonoses, or spillback, from the community. In other studies assessing seroprevalence in companion animals living in households with SARS-CoV-2 infected owners, seropositivity of 4–23.5% has been reported in cats and 13–20% in dogs [12,26,29,30,31]. The current study suggests human-to-animal transmission of SARS-CoV-2 may occur more often for cats than previously recognized.

This study documents CPE in Vero culture of SARS-CoV-2 recovered from an animal naturally exposed to a human with laboratory-confirmed SARS-CoV-2 infection living in the same household. The virus was successfully isolated from swab samples with low Ct values, consistent with what has been reported for virus isolation from human-derived specimens, which has been successful from samples with Ct values under 25 [32] or 10^6^ RNA copies per mL of sample [33]. Additionally, the dog-derived SARS-CoV-2 was passaged three times, and no CPE was noted, despite a reduction in CT value. The role of being persistently infected Vero cells in this context may require further attention. Despite these viral isolations from pets, this study provides no evidence that companion animals play a role in spreading the virus to humans or other animals. Three out of four households with PCR-confirmed cases were multi-pet households, each with two other animals in the household that were negative for SARS-CoV-2 by RT-PCR. All pets in each of these households had SARS-CoV-2 neutralizing antibodies from follow-up serum collections. We did not evaluate the chain or timing of infection, and the study design does not allow for determination of whether human-to-animal or animal-to-animal transmission occurred. Nevertheless, the CDC recommends that people with suspected or confirmed COVID-19 isolate from their pets, just as they would from other members of their household, to reduce the potential for human-to-animal transmission [34]. 

One factor likely contributing to the proportion of household companion animals testing positive for viral RNA or infectious virus is the relatively short window for sampling following human symptoms or diagnosis. Specifically, all four animals meeting case definition by PCR/sequence were sampled within 7 days of owner diagnosis. A study in dogs in Hong Kong found similar results, with SARS-CoV-2 RNA detection in a dog sampled 14 days following initial symptoms of the human COVID-19 patient and one day following confirmed diagnosis [28]. Experimental infection studies indicate that the window of SARS-CoV-2 shedding in cats is up to 7 days [8]. Another factor contributing to the detection of positive animals in the current study is the multiple swab types (three to five) taken from each animal, the two gene targets used for screening all animals, and the two additional gene targets used to confirm presumptive positives. With this approach, we detected viral RNA from the sequential sampling events of two infected cats (TAMU-013 and 057) at least three weeks after their initial positive tests. Prolonged duration of PCR-positivity has also been observed in pharyngeal swabs from post-symptomatic humans recovered from COVID-19 [35]. In that study, although the observed ongoing cellular anti-SARS-CoV-2 response is indicative of persistent viral replication, the studied human cases were not associated with any transmission to close contacts, leading to the interpretation that persistent PCR positive individuals are not contagious [35]. Further, persistent infections have been reported for other coronavirus infections, and the role of persistent infection with SARS-CoV-2 in animals is yet to be fully explored. For example, persistent FCoV shedding has been reported in cats, with shedding lasting >18 months in some cases [36].

There were four detections from body (fur) swabs by PCR. Two of these samples were in the same cat (TAMU-013); the first positive body swab coincided with the initial sampling event when the cat’s respiratory and rectal swabs were also strongly positive, and the second occurred three weeks later when only the body swab was positive. Given the self-licking and grooming behavior of cats, the viral RNA on the fur may reflect self-contamination or reflect living in a household environment where the virus had persisted in prior weeks. The other samples were from two dogs that lived together (household S); one dog also had a positive rectal swab, and the other tested positive only on the body swab; however, neither dog met the case definition. Upon follow-up sampling 3.5 weeks later, both dogs were PCR-negative and remained VN-negative, suggesting they were not infected and the initial detections likely reflected living in a contaminated environment. Attempts to isolate the virus from all four PCR-positive fur samples were unsuccessful, providing further evidence that pet fur is not a fomite for SARS-CoV-2 transmission. 

This study documents that over 25% of the households sampled had pets with neutralizing antibodies. The neutralizing antibody results in this study were based on an inclusive cut-off of 1:8 to consider an animal positive. In general, the titers of neutralizing antibodies in animals presented here seem to be stable or increase over time, with no evidence of seroreversion in the 2–3-month follow-up time period. Several animals initially tested PCR-positive yet VN-negative, followed by seroconversion and then increasing VN titers. More studies evaluating the dynamic of infection not only in pets but also in their respective owners would help elucidate the meaning of neutralizing antibody titer variation in companion animals. This neutralization test was designed to be specific to SARS-CoV-2, and no cross-reactivity between SARS-CoV-2 and type I and II of feline peritonitis virus was detected in a study using similar methods in a cat in Wuhan [12]. Analogously, no cross-reaction between canine endemic coronaviruses and SARS-CoV-2 was observed using virus neutralization and plaque reduction neutralization test in a SARS-CoV-2 positive dog in Italy [11]. Nonetheless, additional serology assays, including other coronaviruses, may be warranted to confirm monotypic reactions to SARS-CoV-2. 

Very few case studies of natural infection in cats and dogs document severe clinical outcomes, and those that have revealed that co-morbidities likely played a contributing factor in illness or death [28,37]. In our study, across all 17 animals that were PCR-positive, VN-positive, or both, only three animals were reported to have mild signs of disease, including 3 days of sneezing in a PCR-positive cat; lethargy in a PCR-positive cat; and sneezing in an antibody-positive dog. Upon re-sample, these and all other animals in the study were reported to be in good health. Based on OIE reports, slightly less than half of all animals reported with SARS-CoV-2 infection have clinical signs, which may include fever, coughing, difficulty breathing or shortness of breath, lethargy, sneezing, nasal discharge, ocular discharge, vomiting, and diarrhea [4]. Our results, which reveal a much higher proportion of asymptomatic animals, suggest that infected companion animals showing no clinical signs or only mild, transient illness may be more numerous than global reporting currently captures. Further active surveillance efforts would more accurately determine the true burden of this virus within the companion animal population.

A study limitation is that it is unknown how many SARS-CoV-2-positive humans lived in each household or the duration or nature of their symptoms while interacting with pets. Nonetheless, our observations of pets with substantive VN titers only days after the owner’s diagnosis (e.g., dog TAMU-062 and cat TAMU-078, which each had VN titers of 128 only 7 and 5 days after the owner’s diagnosis, respectively) suggest that animal infection occurred before human diagnosis. Further, specimens or sequences from the human SARS-CoV-2 infections in the same households were not available for alignment with the sequences we generated from the pets, as the diagnostic labs were not archiving the human samples when testing was complete. Additionally, because animals associated with initial negative results in households with other negative animals were never re-sampled, we may have missed seroconversion at a later date.

The present study advances our understanding of the transmission risk between people and their pets during the COVID-19 pandemic. Moreover, it underscores the need for a One Health approach [38,39], both in epidemiological investigations and in prevention and control measures, as well as pandemic preparedness for SARS-CoV-2 and other emerging zoonotic infectious diseases.

## Figures and Tables

**Table 1:** Pets with positive screening or confirmatory qRT-PCR results for detection of SARS-CoV-2 virus from households with at least one confirmed case of human COVID-19 in Brazos County, Texas, at first (A) and subsequent (B–D) specimen collection visits from June-September 2020.

							Ct Values of qRT-PCR						
Animal ID	Species	Age, Sex, Breed	Household ID	Sampling Order	Days from Human Dx	Swab	RdRp Gene	E Gene	N1 Gene	N2 Gene	Viral Isolation	Viral Neutralization Titer	Animal Symptoms
TAMU-013 *	Cat	3y old male domestic shorthair	E	A	6	Respiratory ‡	21.73	21.12	18	17.7	Positive	<1:8	Sneezing for 3 days reported after initial sampling
						Rectal	34.06	34.26	31.6	32.4	ND		
						Body	34.42	35.03	33.1	32.8	ND		
				B	27	Respiratory	ND	ND	X	X	ND	1:256	None
						Rectal	ND	ND	X	X	X		
						Body	36.88	35.05	ND	ND	ND		
				C	39	All Swabs †	ND	ND	X	X	X	1:512	None
				D	67	All Swabs †	X	X	ND	ND	X	1:2048	None
TAMU-043	Dog	12y old female "Schnoodle"	S	A	9	Respiratory	ND	ND	X	X	X	<1:8	None
						Rectal	ND	ND	X	X	X		
						Body	37.86	37.28	ND	ND	ND		
				B	35	All Swabs †	ND	ND	X	X	X	<1:8	None
TAMU-044	Dog	7y old female Coton de Tulear	S	A	9	Respiratory	ND	ND	X	X	X	<1:8	None
						Rectal	ND	38.72	37.36	39.06	ND		
						Body	37.07	36.18	ND	ND	ND		
				B	35	All Swabs †	ND	ND	X	X	X	<1:8	None
TAMU-057 *	Cat	5y old female domestic shorthair	AA	A	7	Respiratory	35.06	32	ND	37.8	ND	1:128	None
						Rectal ‡	33.4	30.61	33.9	35.5	ND		
						Body	ND	ND	X	X	X		
				B	18	Respiratory	39.53	38.97	ND	ND	ND	1:256	None
						Rectal	ND	ND	X	X	X		
						Body	ND	ND	X	X	X		
				C	32	Respiratory	38.54	ND	ND	ND	X	1:128	None
						Rectal	ND	ND	X	X	X		
						Body	ND	ND	X	X	X		
				D	54	All Swabs †	X	X	ND	ND	X	1:128	None
TAMU-077 *	Dog	5y old female pit bull/bulldog mix	NN	A	6	Respiratory ‡	31.2	30.29	31.1	31.6	Positive	<1:8	None
						Rectal	ND	ND	X	X	X		
						Body	ND	36.56	ND	36.5	X		
				B	19	All Swabs †	ND	ND	X	X	X	1:8	None
				C	37	All Swabs †	X	X	ND	ND	X	1:16	None
TAMU-078 *	Cat	6 mo. old female domestic shorthair	OO	A	5	Respiratory ‡	33.61	32.3	34.7	37.4	ND	1:128	Lethargy reported after initial sampling
						Rectal	ND	ND	X	X	X		
						Body	ND	ND	X	X	X		
				B	17	All Swabs †	ND	ND	X	X	X	1:128	None
				C	38	All Swabs †	X	X	ND	ND	X	1:128	None

RdRp = RNA-dependent RNA Polymerase; E = Envelope; N1/N2 = Virus Nucleocapsid gene target region 1/2; ND = Not detected; X = Not run; * Confirmed positive at NVSL; case definition met based on positive N1 and N2 assays plus sequence confirmation of the virus; † Respiratory (Oral, Nasal, and/or Conjunctival), Body, and Rectal swabs all had no SARS-CoV-2 RNA detected; ‡ Full genome sequence obtained.

**Table 2:** Pets with positive isolation for SARS-CoV-2. The Ct value for RdRp qRT-PCR and viral titer is shown following each of three passages on Vero cells. Work conducted at Biosafety Level 3 Facility at Texas A&M University.

Animal ID	Species	Swab	Original Sample		First Passage		Second Passage		Third Passage	
			RdRp Ct Value	Titer (PFU/mL)	RdRp Ct Value	Titer (PFU/mL)	RdRp Ct Value	Titer (PFU/mL)	RdRp Ct Value	Titer (PFU/mL)
TAMU-013	Cat	Respiratory	21.73	2.3 × 10^3^	15.73	8.1 × 10^3^	15.55	4.9 × 10^4^	15.04	1.1 × 10^5^
TAMU-077	Dog	Respiratory	31.2	ND	ND	ND	33.72	ND	16.2	ND

RdRp = RNA-dependent RNA polymerase; ND = Not detected; Respiratory = Oral, Nasal and/or Conjunctival.

**Table 3:** Pets with SARS-CoV-2 neutralizing antibody titers from households with at least one confirmed case of human COVID-19 in Brazos County, Texas, at first (A) and subsequent (B–D) sampling visits from June-September, 2020. All households with at least one VN-positive animal are shown, with all animals living in the household tested one or more times. For the column of PCR results, any positive PCR result in any of the screening or confirmatory assays is considered positive.

Household ID	Animal ID	Species	Age, Sex Breed	Sampling Order	Days from Human Dx	PCR Positive Samples	Virus Neutralization Titer
D	TAMU-009	Cat	4y old male domestic shorthair	A	27	0	1:128
				B	93	0	1:64
	TAMU-010	Cat	5y old male domestic shorthair	A	27	0	1:64
				B	93	0	1:128
E	TAMU-011	Dog	5y old male red lacie	A	6	0	<1:8
				B	27	0	1:128
	TAMU-012	Dog	3y old male aussie/border collie mix	A	6	0	1:16
				B	27	0	1:16
				C	77	0	1:8
	TAMU-013	Cat	3y old male short haired tabbie	A	6	Resp., Rectal, Body	<1:8
				B	27	Body	1:256
				C	39	0	1:512
				D	67	0	1:2048
K	TAMU-027	Dog	2y old male huskie/border collie mix	A	10	0	<1:8
	TAMU-028	Dog	2y old female huskie/border collie mix	A	10	0	1:16
				B	66	0	1:8
	TAMU-029	Dog	5y old female bluenose pit bull	A	10	0	<1:8
				B	66	0	<1:8
	TAMU-030	Dog	2y old male rotweiler	A	10	0	<1:8
				B	66	0	<1:8
N	TAMU-034	Cat	Female domestic shorthair (unknown age)	A	8	0	1:64
O	TAMU-035	Dog	12y old male dachshund	A	8	0	1:32
W	TAMU-048	Dog	Male Siberian husky (unknown age)	A	12	0	1:64
AA	TAMU-055	Dog	6.5y old female shitzu/chihuahua mix	A	7	0	1:64
				B	18	0	1:32
				C	32	0	1:64
				D	54	0	1:8
	TAMU-056	Dog	18y old male pit bull/pointer mix	A	7	0	1:16
				B	18	0	1:16
				C	32	0	1:64
				D	54	0	1:32
	TAMU-057	Cat	5y old female tabby	A	7	Resp., Rectal	1:128
				B	18	Resp.	1:256
				C	32	Resp.	1:128
				D	54	0	1:128
FF	TAMU-062	Dog	5y old male German shepherd	A	7	0	1:128
NN	TAMU-077	Dog	5y old female put bull/bulldog mix	A	6	Resp., Body	<1:8
				B	19	0	1:8
				C	37	0	1:16
OO	TAMU-078	Cat	6mo old female domestic shorthair	A	5	Resp.	1:128
				B	17	0	1:128
				C	38	0	1:128
	TAMU-079	Cat	6mo old female domestic shorthair	A	5	0	1:64
				B	17	0	1:32
				C	38	0	1:64
	TAMU-080	Cat	6mo old female domestic shorthair	A	5	0	1:64
				B	17	0	1:64
				C	38	0	1:16

## Data Availability

Full genomes generated in this study were submitted to Genbank (accession nos. MW263334-7).
